# Individual characteristics, area social participation, and primary non-concordance with medication: a multilevel analysis

**DOI:** 10.1186/1471-2458-6-52

**Published:** 2006-03-02

**Authors:** Kristina Johnell, Martin Lindström, Jan Sundquist, Charli Eriksson, Juan Merlo

**Affiliations:** 1 Centre for Family Medicine, Karolinska Institutet, Stockholm, Sweden; 2 Community Medicine (Dept Clin Sci), Malmö University Hospital, Faculty of Medicine, Lund University, Sweden; 3 Department of Community Medicine and Public Health, Regional Medical Centre, Örebro, Sweden

## Abstract

**Background:**

Non-concordance with medication remains a major public health problem that imposes a considerable financial burden on the health care system, and there is still a need for studies on correlates of non-concordance. Our first aim is to analyse whether any of the individual characteristics age, educational level, financial strain, self-rated health, social participation, and trust in the health care system are associated with primary non-concordance with medication. Our second aim is to investigate whether people living in the same area have similar probability of primary non-concordance with medication, that relates to area social participation.

**Methods:**

We analysed cross sectional data from 9 070 women and 6 795 men aged 18 to 79 years, living in 78 areas in central Sweden, who participated in the Life & Health year 2000 survey, with multilevel logistic regression (individuals at the first level and areas at the second level).

**Results:**

Younger age, financial strain, low self-rated health, and low trust in the health care system were associated with primary non-concordance with medication. However, area social participation was not related to primary non-concordance, and the variation in primary non-concordance between the areas was small.

**Conclusion:**

Our results indicate that people in central Sweden with younger age, financial difficulties, low self-rated health, and low trust in the health care system may have a higher probability of primary non-concordance with medication. However, the area of residence – as defined by administrative boundaries – seems to play a minor role for primary non-concordance.

## Background

Patients' concordance with medication is a prerequisite for effective drug therapy. Non-concordance is a major public health problem that imposes a considerable financial burden on the health care system [1,2]. Despite the comprehensive research on concordance during the last decades [3], non-concordance remains a concern in health care, and there is still a need for studies on correlates of non-concordance because the complex phenomenon of non-concordance is far from understood. It has been suggested that the social context in which non-concordance occurs should not be ignored [1,2,4], and we have tried to incorporate this aspect in this study.

The term concordance implies agreement, trust, and harmony between patient and doctor regarding treatment, and acknowledges the patient as a decision maker, and a cornerstone is professional empathy [2,5]. The members of a working party of the Royal Pharmaceutical Society of Great Britain introduced the term concordance, which recognizes a patient's own choice to concord with treatment [2].

Patient non-concordance with medication may be divided into primary non-concordance, where the patient does not redeem the prescription, and secondary non-concordance, where the patient does not take the medication as prescribed [2,6]. Most studies have focused on secondary non-concordance [7-9]. Nevertheless, it is crucial to determine whether patients actually redeem their prescriptions from the pharmacy, because this is the first step in the complex phenomenon of concordance [9]. Studies on primary non-concordance have reported non-redemption rates between 2% and 33% [6-16]. However, these studies vary greatly regarding assessment of primary non-concordance, participants, and setting.

Individual characteristics, such as age, educational level, self-rated health, and social support have been discussed as correlates of concordance, however, the results are inconsistent [1-3,17,18]. On the other hand, the influence of area factors, related to one's area of residence, have been scarcely investigated in relation to concordance. Yet, over and above individual characteristics, patients' concordance with medication might be related to the social context in which they live [1,4]. In a previous study, we observed that the association between social participation and concordance with antihypertensives varied among municipalities in Scania, Sweden (i.e., cross-level interaction) [19], which suggests that the area of residence may influence the mechanisms behind the concordance behaviour.

Individual social participation describes how actively a person takes part in activities, groups, and associations, and social participation has been associated with health-related behaviours, such as smoking cessation [20] and physical activity [21]. Further, social participation is important for understanding the influence of social factors on health [22], and can be viewed as a feature of individual social networks [23]. Social participation and social networks have been suggested to influence health behaviours, such as concordance with medication, possibly through information exchange and establishment of health-related group norms [23,24]. In our previous study in Scania, our results suggested an association between low social participation and low concordance with antihypertensives [19], and, therefore, we wanted to further investigate whether social participation, both at the individual and at the area level, was associated with general primary non-concordance with medication, in a different setting.

The area level of social participation has been considered as a structural component within the concept of social capital [24,25], which describes social structures and social relationships in society [24]. Living in an area with low social capital might decrease the individual probability of concordance with medication through mechanisms like poorer social networks [17], shared norms around health-related behaviour, transmission of health information, health care system factors [26], and social control over deviant health-related behaviour [23,27]. Previous studies have found associations between living in a disadvantaged area [28], with low social capital [29,30], and use of medication. We were therefore interested in investigating whether social capital, as measured by the area level of social participation, might be related to primary non-concordance with medication.

Because of our contextual approach in this study, we used multilevel analysis, which handles information on both people and context simultaneously within the same model [31], and we investigated measures of variation as well as traditional measures of association [32,33].

The first aim of this study is to analyse whether any of the individual characteristics age, educational level, financial strain, self-rated health, social participation, and trust in the health care system are associated with primary non-concordance with medication. The second aim is to investigate whether people living in the same area have similar probability of primary non-concordance with medication, that relates to area social participation.

## Methods

### Study sample

We used data from the Life & Health year 2000 survey [34], a postal questionnaire administered by Statistics Sweden. A random sample of 70 044 people, aged 18–79 years from 58 municipalities in six regions in central Sweden (Södermanland, Uppsala, Värmland, Västmanland, and Örebro county, and south Dalarna), had the opportunity to participate in the survey and 46 636 (67%) returned the questionnaire. The purpose of the survey was to generate self-reported information about people's life and health in the area and was complemented with register data on age, sex, place of residence, and educational level. Of the 46 636 participants, we included those who reported having visited an emergency department, a physician at a hospital department, a primary care physician, or been admitted to a hospital during the last 3 months (n = 20 362), and with complete information on all the variables studied (n = 9 070 women and 6 795 men).

The areas in this study correspond to municipalities, except for Uppsala, Västerås, and Örebro (the three largest cities in the sample region), which were divided into ten, eight, and five smaller urban areas, respectively. In total, there were 78 areas.

### Outcome variable

*Primary non-concordance with medication *(dichotomous) was assessed by the question "During the last 3 months, have you received a prescription for medicine, but not redeemed the medicine?"

### Explanatory variables

The correlates of non-concordance were selected by reviewing the literature and grouped into a) social and economic factors and b) Health care system and area factors, as suggested by the World Health Organization (WHO) [26].

#### Social and economic factors

*Age *was categorized into four groups: 18–34 years, 35–49 years, 50–64 years, and 65–79 years (used as reference category).

*Educational level *was dichotomised into ≥9 years of education (i.e., compulsory school) (low educational level) and >9 years [35].

*Financial strain *was assessed by a negative answer to the question, "Would you manage to raise 18 000 SEK (about 1900 Euro) in 1 week?" (dichotomous).

*Self-rated health *was assessed by the question "How do you rate your general health status?" and dichotomised into "neither good nor bad/bad/very bad health" (low self-rated health) and "good/very good health" [36].

*Individual social participation *was defined by active membership [37] in a labour union, political party, council/board, community centre, sports association, cultural association/choir/orchestra/theatre group etc., religious association/community, or other association. Participants without any active membership in any of these associations were considered to have low social participation (dichotomous).

#### Health care system and area factors

*Trust in the health care system *was assessed by the question "How much do you trust the following institutions in society?" and for "The health care system" indicating "not particularly high trust/no trust/have no opinion" (low trust in the health care system) and "very high trust/fairly high trust" (dichotomous).

*Area low social participation *was based on a larger sample from the Life & Health year 2000 survey (20 715 women and 18 190 men), and was estimated by the proportion of participants in the area who were classified as having low social participation [29,30,37]. Area low social participation was then divided into tertiles.

### Statistical analysis

We used multilevel logistic regression analysis [38] with individuals at the first level and areas at the second level. Men and women were analysed separately, in order to see whether the associations between the explanatory variables and the outcome were different for men and women.

In Model i (empty model), we did not include any explanatory variables. In Model ii, we included only the individual variables, i.e., age, education, financial strain, self-rated health, social participation, and trust in the health care system. In Model iii, we added area low social participation, because we wanted to investigate the influence of area low social participation on primary non-concordance with medication, after adjustment for the individual (compositional) variables.

#### Fixed effects (measures of association)

The results are shown as odds ratios (ORs) with 95 % confidence intervals (CIs).

#### Random effects (measures of variation)

We examined whether the area of residence had a general contextual effect on individual primary non-concordance. In other words, we wanted to establish whether individuals living in the same area shared a similar probability of primary non-concordance, after adjusting for the individual characteristics studied. This hypothesized contextual phenomenon [33] was measured by the intraclass correlation (ICC) and the median odds ratio (MOR). We also applied the 80% interval odds ratio (80%IOR), which integrates random effects (i.e., area variance) in the measurement of fixed effects (i.e., the area variable) [38,39]. We refer elsewhere[38,39] for a more detailed explanation of the ICC, MOR and IOR. However, in short, the ICC is the proportion of the total variance (i.e., the variance at the area level plus the variance at the individual level) that is at the area level. In the multilevel logistic regression, there are different ways to calculate the ICC [40]; however, we chose the threshold model, as described by Snijders & Bosker [41]. An advantageous alternative to the ICC in the multilevel logistic regression is the MOR, which measures area variance in the odds ratio scale. If the MOR is equal to 1 (no area level variance), there is no difference between the areas regarding primary non-concordance. Conversely, the higher the MOR, the more important the contextual effects for understanding the individual probability of primary non-concordance.

The IOR considers the magnitude of the difference between the areas regarding primary non-concordance when interpreting the influence of area variables. It has been suggested to report the IOR as an 80% interval, and also, the IOR is not an ordinary confidence interval. If the IOR contains 1, the remaining unexplained difference between the areas regarding primary non-concordance is large compared with the effect of the area variable. However, if the IOR does not contain 1, the effect of the area variable is large compared with the unexplained difference between the areas.

The MLwiN software, version 2.0 [42], was used for the analyses. Parameters were estimated using the Markov Chain Monte Carlo (MCMC) procedure. We used the default settings in MLwiN, i.e., chains of length 5 000 after a burn-in of 500. The Deviance Information Criterion (DIC) was used as a measure of how well our different models fitted the data. A lower value on DIC indicates a better fit of the model [43,44].

## Results

The prevalence of primary non-concordance with medication was 7.6% (range across the 78 areas: 2%–14%) for women and 6.5% (range across the 78 areas: 0%–15%) for men in the study sample. Financial strain and low social participation were more frequent among women than among men (table 1).

Further, table 1 shows the distribution of active membership in associations (social participation) in the study sample. Active membership in sports associations was fairly frequent (i.e., in 8.9% of women and 15.5% of men). However, a large proportion of the participants were members of "other", unknown, associations (i.e., 22.7% of women and 26.5% of men).

The median (first to third quartile) number of participants in the areas was 115 (102–126) women and 86 (72–94) men. Area low social participation ranged from 45% to 63%.

### Fixed effects

#### Social and economic factors

In Model ii, the association between age and primary non-concordance with medication was inversed (table 2 and 3). Moreover, we did not find any association between educational level and primary non-concordance, but we found an association between financial strain and primary non-concordance (OR_women _= 1.87 (95% CI 1.58–2.22) and OR_men _= 2.24 (95% CI 1.82–2.75)). Also low self-rated health (OR_women _= 1.38 (95% CI 1.16–1.63) and OR_men _= 1.58 (95% CI 1.28–1.95)) was associated with primary non-concordance. The lower DIC value in Model ii indicated a better fit than in Model i (the empty model).

#### Health care system and area factors

In Model ii, low trust in the health care system ((OR_women _= 1.33 (95% CI 1.12–1.58) and OR_men _= 1.37 (95% CI 1.11–1.69)) was related to primary non-concordance.

In Model iii, area low social participation was not associated with primary non-concordance. Furthermore, the DIC value in Model iii showed that the addition of area low social participation did not improve the fit.

### Random effects

The area intercept variance in the different analyses was small, ranging from 0.012 to 0.036 (table 2 and 3). Accordingly, the MOR and the ICC were also small, the MOR ranging from 1.11 to 1.20 and the ICC ranging from 0.4% to 1.1%.

Moreover, the 80%IORs included 1 for area low social participation, and thereby confirmed the low importance, in this study, of this area characteristic for primary non-concordance.

We did not find any interaction between individual and area social participation.

## Discussion

### Main findings

Our results indicate that the individual characteristics younger age, financial strain, low self-rated health, and low trust in the health care system are associated with primary non-concordance with medication. Our finding that younger age is associated with higher primary non-concordance is in line with previous studies on primary non-concordance [10,13,15].

Research on education and concordance has been inconclusive [1,18], and we did not either find an association between educational level and primary non-concordance. However, there was a relation between financial strain and primary non-concordance. All residents in Sweden who have spent 1800 SEK (about 190 Euro) on medication within the preceding 12 months are entitled to free prescribed medicines through the social security system [45]. Despite this subsidy, participants in this study who experienced financial strain seemed to have difficulties redeeming their medication. It has previously been reported that financial barriers contribute to non-concordance [3,7,12,13,45-47], and that people who restrict their use of medication because of cost have worse health outcomes [48].

Also, we observed an association between low self-rated health and primary non-concordance. However, previous studies on self-rated health and concordance have given different results [18].

Furthermore, we found a relation between low trust in the health care system and primary non-concordance. There is an ongoing discussion about the lack of people's trust in health care system and health care professionals [49-55]. It is known that trust and the quality of the doctor-patient relationship are important for concordance with medication [2,51,56-58]. Possible ways to enhance patient trust may be continuity of care from a regular doctor [59] and provision of patient centred care where the patient gets enough attention [60]. Future studies of why some people have low trust in the health care system and how this trust can be enhanced are needed.

Further, area social participation was not associated with primary non-concordance with medication. The low importance of the area is further supported by the low area variances and, hence, the low MORs in the analyses, which indicate that the area of residence, as measured in this study, does not seem to be important for primary non-concordance. Nevertheless, one alternative explanation could be that the choice of areas in this study as municipalities and the way we measured the aggregated variable area social participation did not capture the context important for primary non-concordance. We expected area social participation, a main aspect of social capital, to be related to primary non-concordance with medication because of mechanisms like poorer social networks, shared norms around health-related behaviour, transmission of health information, and social control over deviant health-related behaviour [23,27]. Indeed, related research suggests that social capital and deprivation plays a role in use of medication [28,30], how health care is perceived by citizens [61] and people's trust in their physicians may be influenced by contextual variables [62].

### Limitations

The study sample consisted of those who had visited an emergency department, a physician at a hospital department, a primary care physician, or been admitted to a hospital. With this definition of population at risk we tried to include individuals that had had some contact with the health care system and therefore could have received a prescription. However, this definition may not adequately capture people "at risk" of not redeeming a prescription for medicine.

We used self-reported concordance, which has been found to correlate with other measures of concordance and with clinical measures of disease activity [63-65]. Further, self-report offers a convenient and non-invasive estimate of concordance behaviour. However, the procedure of measuring concordance is controversial. Self-report can be subject to self-presentational and recall biases. People may overestimate their concordance and their memory may be inaccurate [66].

The WHO has suggested five sets of correlates of non-concordance: social and economic factors, health care system factors, condition-related factors, therapy-related factors, and patient-related factors [26]. We have only addressed two of these five sets of correlates: social and economic factors and health care system and area factors. Future studies of non-concordance may try to also capture the less often studied condition-related factors, such as severity of symptoms, and therapy-related factors, such as the complexity of the medical regimen.

People living in deprived areas and with low socio-economic status, low self-rated health, and primary non-concordance with medication may have been less inclined to respond to the Life & Health year 2000 survey. Analyses of the excluded responders with incomplete information on the variables studied showed that they were, in general, older, and had lower educational level and lower self-rated health, than those included in this study. This possible selection bias could lead to an underestimation of the associations between the explanatory variables in this study and the outcome primary non-concordance with medication.

The cross-sectional design of this study is a weakness, because the direction of causality is impossible to determine. However, the direction of causality from individual characteristics to primary non-concordance seems to be the most plausible.

## Conclusion

Our results indicate that people in central Sweden with younger age, financial difficulties, low self-rated health, and low trust in the health care system may have a higher probability of primary non-concordance with medication. However, the area of residence – as defined by administrative boundaries – seems to play a minor role for primary non-concordance.

Future studies of why some people have low trust in the health care system and how this trust can be enhanced are needed.

## Competing interests

The author(s) declare that they have no competing interests.

## Authors' contributions

KJ and JM developed the original idea, participated in the design of the study, performed the statistical analyses, and drafted the manuscript. ML and JS participated in the design of the study and revised the manuscript. CE participated in the design of the study, helped to collect the data, and revised the manuscript. All authors read and approved the final manuscript.

## Pre-publication history

The pre-publication history for this paper can be accessed here:



## Figures and Tables

**Table 1:** Characteristics of the 9 070 women and 6 795 men from the Life & Health year 2000 survey, Sweden. Unless otherwise indicated, values are given in percent.

	Women	Men
Mean age	49 years	53 years

Primary non-concordance with medication	7.6	6.5
Low educational level	30.6	33.9
Financial strain	33.7	24.2
Low self-rated health	45.9	44.2
Low social participation	54.9	47.5
Low trust in the health care system	26.2	25.1
Active membership in associations (social participation)		
Labour union	4.1	4.7
Political party	2.2	3.2
Council/board	2.9	2.7
Community centre	3.0	4.0
Sports association	8.9	15.5
Cultural association/choir/orchestra/theatre group, etc.	7.9	7.7
Religious association/community	6.1	4.5
Other association	22.7	26.5

**Table 2:** Area variance and odds ratios (ORs) with 95% confidence intervals (95% CIs) for individual variables and area low social participation regarding primary non-concordance with medication among 9 070 women from the Life & Health year 2000 survey, Sweden.

	Model i (empty model)		Model ii		Model iii		
	OR	95% CI	OR	95% CI	OR	95% CI	80% IOR

*Fixed effects*							
Age							
65–79 years			Ref		Ref		
50–64 years			1.58	(1.16–2.15)	1.55	(1.18–2.03)	
35–49 years			2.49	(1.82–3.43)	2.46	(1.88–3.22)	
18–34 years			3.22	(2.36–4.39)	3.17	(2.41–4.17)	
Low educational level (yes vs. no)			0.85	(0.71–1.03)	0.85	(0.71–1.03)	
Financial strain (yes vs. no)			1.87	(1.58–2.22)	1.85	(1.57–2.18)	
Low self-rated health (yes vs. no)			1.38	(1.16–1.63)	1.37	(1.16–1.62)	
Low social participation (yes vs. no)			0.87	(0.74–1.02)	0.86	(0.73–1.02)	
Low trust in the health care system (yes vs. no)			1.33	(1.12–1.58)	1.34	(1.13–1.58)	
Area low social participation in tertiles							
1					Ref		
2					1.22	(0.99–1.50)	0.98–1.51
3					1.10	(0.89–1.35)	0.88–1.36
							
*Random effects*							
Area intercept variance (SE)	0.014(0.015)		0.012(0.013)		0.014(0.015)		
Median odds ratio	1.12		1.11		1.12		
Intraclass correlation	0.4%		0.4%		0.4%		
							
Deviance information criterion	4888		4686		4686		

**Table 3:** Area variance and odds ratios (ORs) with 95% confidence intervals (95% CIs) for individual variables and area low social participation regarding primary non-concordance with medication among 6 795 men from the Life & Health year 2000 survey, Sweden.

	Model i (empty model)		Model ii		Model iii		
	OR	95% CI	OR	95% CI	OR	95% CI	80% IOR

*Fixed effects*							
Age							
65–79 years			Ref		Ref		
50–64 years			1.57	(1.18–2.09)	1.57	(1.17–2.09)	
35–49 years			2.23	(1.64–3.04)	2.19	(1.61–2.98)	
18–34 years			2.29	(1.67–3.13)	2.26	(1.62–3.16)	
Low educational level (yes vs. no)			0.87	(0.69–1.10)	0.87	(0.70–1.09)	
Financial strain (yes vs. no)			2.24	(1.82–2.75)	2.25	(1.84–2.76)	
Low self-rated health (yes vs. no)			1.58	(1.28–1.95)	1.58	(1.29–1.93)	
Low social participation (yes vs. no)			0.85	(0.69–1.03)	0.85	(0.69–1.03)	
Low trust in the health care system (yes vs. no)			1.37	(1.11–1.69)	1.38	(1.11–1.72)	
Area low social participation in tertiles							
1					Ref		
2					1.07	(0.80–1.43)	0.76–1.51
3					0.83	(0.62–1.11)	0.59–1.17
							
*Random effects*							
Area intercept variance (SE)	0.034(0.033)		0.034(0.033)		0.036(0.025)		
Median odds ratio	1.19		1.19		1.20		
Intraclass correlation	1.0%		1.0%		1.1%		
							
Deviance information criterion	3288		3148		3148		
